# Older adults as designers of behavior change strategies to increase physical activity—Report of a participatory design process

**DOI:** 10.3389/fpubh.2022.988470

**Published:** 2022-12-21

**Authors:** Rebecka Janols, Marlene Sandlund, Helena Lindgren, Beatrice Pettersson

**Affiliations:** ^1^Department of Community Medicine and Rehabilitation, Occupational Therapy, Umeå University, Umeå, Sweden; ^2^Department of Computing Science, Umeå University, Umeå, Sweden; ^3^Department of Community Medicine and Rehabilitation, Physiotherapy, Umeå University, Umeå, Sweden

**Keywords:** participatory design, co-creation, behavior change, motivation, digital technology, mHealth

## Abstract

**Background:**

Despite the significant value of physical activity for the health of older adults, this population often fails to achieve recommended activity levels. Digital interventions show promise in providing support for self-managed physical activity. However, more information is needed about older adults' preferences for digital support to change physical activity behaviors as well as the process of designing them. The aim of this paper was to describe the participatory design process in which older adults were involved in the co-creation of digitally supported behavioral change strategies to support self-managed physical activity, and how the results were integrated in a prototype.

**Methods:**

The participatory design process involved with nine older adults and two researchers. The participants were divided in two groups, and each group participated in three workshops and completed home tasks in between workshops. Following an iterative design process influenced by theories of behavior change, the workshops and home tasks were continuously analyzed, and the content and process were developed between groups and the next set of workshops. Prototypes of a mobile health (mHealth) solution for fall preventive exercise for older adults were developed in which the conceptualized strategies were integrated. To support coherence in reporting and evaluation, the developed techniques were mapped to the Behavior Change Technique Taxonomy v1 and the basic human psychosocial needs according to the Self-determination Theory.

**Results:**

The results highlight different preferences of older adults for feedback on physical activity performance, as well as the importance of transparency regarding the identification of the sender of feedback. Preferences for content and wording of feedback varied greatly. Subsequently, the design process resulted in a virtual health coach with three different motivational profiles and tools for goal setting and self-monitoring. These behavior change strategies were integrated in the exercise application Safe Step v1. The conformity of the design concepts with the needs of Self-determination Theory and Behavior Change Technique Taxonomy v1 are presented.

**Conclusion:**

The participatory design process exemplifies how older adults successfully contributed to the design of theory-based digital behavior change support, from idea to finished solution. Tailoring feedback with a transparent sender is important to support and not undermine motivation.

## 1. Introduction

Keeping physically active while aging is important for both physical and mental health (1). Physical activity can prevent accidental falls and non-communicable diseases such as type 2 diabetes and coronary heart disease (2, 3). However, older adults who are independently living spend on average 9.4 h of their waking day sedentary (4). A recent meta-analysis reported a significant relationship between sedentary behavior and all-cause mortality, showing an increased risk of mortality from about 7.5–9 h of sedentary time, with an increasing risk above 9.5 h. According to physical activity guidelines (3), older adults should do at least 150 min per week of moderate-intensity aerobic activity or at least 75 min of vigorous-intensity activity, balance and strength exercises at least three times per week, and limit time spent sedentary. However, in a UK population, only 10% of women and 15% of men over the age of 65 reached the recommended levels of physical activity (5). Therefore, greater efforts need to be made to support older adults in meeting recommendations for physical activity and to reduce sedentary behavior (3, 5). Unfortunately, interventions which aim to support community-dwelling older adults in physical activity or exercise often fail to enhance long-lasting engagement (6). There is, therefore, a need for new ways to support changes in physical activity.

Emerging evidence supports the use of digital technologies, such as applications for smartphones and tablets, as a way of supporting older adults in increasing their levels of physical activity and decreasing sedentary time (7, 8). The use of mobile health (mHealth) applications show promise in vastly increasing the reach for physical activity interventions to a wider population and provide rapid implementation. mHealth interventions also have the potential to be economically beneficial as older adults can be supported in self-managing their physical activity and health from their own homes with reduced use of health care resources.

Even though mHealth interventions show promise in providing numerous with support for physical activity, many older adults may still need supervision while exercising (9), or face barriers in the adoption of digital devices (10). Therefore, a variety of interventions to support physical activity behavior is needed to meet different preferences and needs of older adults. Still, the use of smart technology is rapidly increasing in this older age group. In Sweden, for example, 75% of older adults report regularly accessing the internet on a smartphone, and 43% using a tablet (11). Even though age is a dominant factor among non-users of the Internet (3.4%), this number is rapidly decreasing (12).

Even though the use of mHealth for self-management of physical activity seems promising for older adults, there are indications that further efforts need to be made to support long-term engagement (8, 13, 14). Incorporating behavior change theories in physical activity interventions and digital technologies has proven effective in supporting physical activity behaviors (15). Nevertheless, overviews of the area show that the behavioral change strategies that are used in applications for physical activity are often few and have not been developed systematically using theoretical frameworks (16, 17). Despite the established importance of incorporating behavior change strategies in digital and non-digital behavior change and physical activity interventions, reporting of such strategies is often poor (17, 18). To further enable comparisons between studies, more thorough descriptions of the development and use of behavior change strategies have been advocated (18, 19).

Involving potential users in the design process of interventions can help to increase ownership of the solutions. Participatory design methodologies can produce valued outcomes by capturing participants' diverse attitudes, needs, preferences, and motivation. To be utilized, technologies need to be perceived as both supportive to the individual in their daily life and as easy to use (20). By involving older adults as “co-designers” in the design process of the digital technology, a better understanding of their requirements is gained and can enhance usability, user experience, and user acceptance (21, 22). Previous research on participatory design of digital technology for older adults has focused on device (re-)design and prototype development, and prototype testing. There is, however, a lack of research that involves older adults from the stage of idea generation and conceptualization (23). Additionally, the participatory design process is rarely described in the research literature. The aim of this paper is therefore to describe the participatory design process in which older adults were involved in the co-creation of digitally supported behavior change strategies to support self-managed physical activity, and how the results were integrated in a prototype.

## 2. Methods

In order to gain a deeper understanding of older adults' motivation for physical activity with support from digital technology, a participatory design process was applied (24). The design process involved six workshops with nine older adults and two researchers, individual home tasks between the workshops, and analysis of the findings to adapt the further design process. Two researchers (one expert within usability and user-centered methods and one physiotherapist) facilitated the process. The study was also part of a larger participatory design process where focus had earlier been on the physical exercise modules of the prototype.

### 2.1. Participants

The criteria for inclusion were community-dwelling older adults (over 70 years of age) with a good understanding of the Swedish language, comprehension of written information and the ability to actively participate in all of the planned workshop activities. To create a heterogeneous group (with regards to age, sex, background, experiences of physical activities and digital technology), the participants were recruited using a convenience sampling approach. A total of nine older adults (four men and five women) aged between 71 and 87 years were included in the present study (Table 1). Four participants were recruited *via* presentations at two senior citizen organizations, three by using the snowball method, and two had expressed interest at an earlier stage of development when other aspects of the prototype were being focused on, but did not participate then.

**Table 1:** Participant characteristics presented by group.

**Group**	**Sex**	**Age**	**Exercise habits**	**Experience of smart technology**
1	F	72	Walking (daily on treadmill)	Yes, tablet
1	M	76	Walking (3/week), Group exercise class	No (regular mobile phone)
1	F	80	Walking (3–5/week), Group exercise class	No (computer and regular mobile phone)
1	F	87	Walking (daily), Aqua aerobics	Yes, smartphone and tablet
2	F	76	Walking (daily), Group exercise class	No (regular mobile phone)
2	M	77	Dog walking (daily), Aqua aerobics	Yes, smartphone
2	M	78	Aqua aerobic, Strength exercises	Yes, smartphone and tablet
2	F	83	Dog walking (daily), Aqua aerobics	Yes, smartphone and tablet
2	M	84	Dog walking (daily), Aqua aerobics	Yes, smartphone and tablet

F, female; M, male.

### 2.2. Workshops

#### 2.2.1. Theoretical foundation

Self-determination Theory (SDT) was chosen as a theoretical framework to guide the intervention and development of the behavior change strategies due to its empirical basis for examining sustained motivation and well-being (25). In SDT, three basic human psychological needs that can enhance or undermine intrinsic motivation are specified: autonomy, competence, and relatedness. When these three needs are satisfied they enhance self-motivation (intrinsic motivation). However, if the needs are neglected or frustrated they can lead to negative experiences and decreased motivation. Autonomy refers to the feeling of volition and choice, to be able to act according to your own personal goals and values while competence refers to a feeling of effectiveness when interacting with other people and obtaining desired outcomes. Relatedness reflects the need of belonging and feeling connected to other people or a context (e.g., to be part of a social context and care and feel cared for by other people) (25). To support all three basic human psychological needs is important for engagement in physical activity. In a study by Teas et al. (26), simply to engage in physical activity was not enough to improve a sense of wellbeing among older adults. The activities also needed to be supportive of all of the psychological needs (26). A meta-analysis confirmed the importance of incorporating elements of all three needs to support physical activity behavior (27).

#### 2.2.2. Design and analysis

A total of six workshops were conducted; three workshops with each group of two researchers and five and four participants, respectively. The participants were allocated to their groups by a member in the research project. Smaller group sizes were chosen to achieve a creative and familiar environment in accordance with recommendations for focus group composition (28). Each group met three times for 2 h on each occasion, including a 15 min coffee break. The workshops were inspired by the method of Participatory Action Research process based on theories on Behavior Change and Persuasive technology (PAR-BCP) (29). The PAR-BCP method can be used to design digital behavior change systems, and the design process involves concepts of tailoring, behavior change, and persuasive systems design (29). The workshops were framed toward appreciative knowledge sharing rather than focusing on “problem fixing” (30). Specific behavior change techniques served as themes for the workshops and were chosen based on experience within the research group as well as evidence of effective behavior change techniques to support physical activity (31). The workshops followed an iterative process in which the content of the workshops and intervention development were adapted by the researchers based on the results of the previous workshop and a home task. The home task was related to continue to reflect on the theme of the previous workshop and created a basis for the researchers' analysis and planning of the next workshop (Table 2). Adapting the content of the workshops was a way to show the participants that their thoughts and reflections were important, a method to create a feeling of ownership. For workshop 1, the choice of theme was intended to help inform the researchers of the participants' experiences and attitudes toward physical activity and digital technology. The theme was also seen as an opportunity to create a positive climate and discussion when moving forward to following workshops (30). One or two interactive activities were undertaken in each workshop (lasting 20–40 mins) and were led by one of the two researchers. Some activities were conducted in smaller sub-groups of 2–3 participants and were followed by a group discussion (Table 2). All workshops and sub-group activities were audio recorded. After each workshop the two researchers discussed the progress of the workshops and key points made by the participants. The discussions were also audio recorded and included in the analysis.

**Table 2:** A summary of the aim of each workshop (WS) and the activities that were performed according to the PAR-BCP methodology (29).

**WS**	**Aim**	**Themes and activities**
1st	Understand the participants' experiences and attitude toward exercise and digital technology (phones/tablets).	**Theme: motivation for physical activity and technology** – Brainstorming: motivation for and experiences of exercise – Post-it notes: individually write what you do/want to do with smart phones and tablets. Followed by group discussion.
HT		**Theme: feedback** Write the messages you want to hear when you have or have not performed planned exercise.
2nd	Build knowledge about what enhances and decreases older adults' motivation to do exercise and how to personalize feedback to support the individuals.	**Theme: feedback** – Digital survey: ranking of feedback messages. – Discussion: interpretation of the content of the feedback messages.
HT		**Theme: technology usage** Write a narrative about how to communicate through and interact with the mHealth application.
3rd	Build knowledge about how to summarize and display results and performance in the mobile application.	**Theme: visualization of performance** – Pictures/cards: Discuss in pairs what the pictures means to you and how they can be used in the presentation of exercise performance. – Select and present your favorites to the group.
	Learn what older adults find important when they meet health professionals.	**Theme: feedback** – Brainstorming: Based on experiences with health professionals, create 3–5 profiles with different personalities.

Between each WS, the participants were asked to do home tasks (HT).

**Figure 1:**
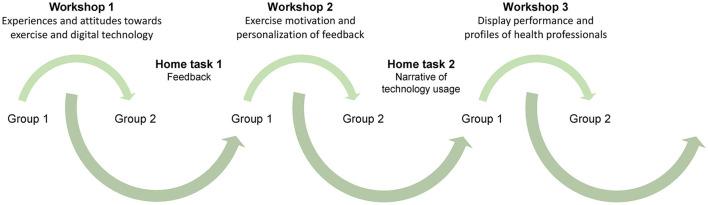
The iterative design process, represented by arrows, including both participant groups and each workshop with analysis between sessions.

Following an iterative process, analysis was performed both between the workshops and after all workshops were conducted (Figure 1). After the first workshop with Group 1, the process was refined and lessons learned were used in the first workshop with Group 2. After both the first and the second workshop, two of the researchers analyzed the process, the collected data, and the home task in order to adapt the process and tailor the content to the participants' interests and the early findings. For example, the results from the first home task “writing feedback messages” were analyzed by the two researchers and used as a basis for activities in both workshop 2 and workshop 3 (Table 2).

Prototypes of a mHealth application were developed in which the conceptualized strategies were integrated, and which aimed at reducing risk of falls for community-living older people through balance and strength training. The resulting Safe Step application v1 presented in this article has been developed at Umeå University, Sweden, iteratively in different phases. In an earlier phase, 17 older adults have been involved in a co-creation process focussing on the exercise modules (32, 33).

To support comparisons between studies, the behavior change strategies developed in this study were analyzed using two analytical matrixes: the three basic human psychological needs of the STD (25) and the Behavior Change Technique Taxonomy v1 (19). After all workshops were completed, the recordings were listened to and content related to the analytical matrixes were transcribed, deductively coded, and clustered by the first and last author. The deductive coding was continuously discussed among all authors. The Behavior Change Technique Taxonomy v1 was developed to support coherence in reporting and facilitating comparisons between interventions (19). The taxonomy consists of 93 defined behavior change techniques, divided into 16 groups. The taxonomy presents techniques for behavior change, but is not underpinned by a specific behavior change theory.

## 3. Results

The results include a description of the findings of the workshops and the resulting prototypes as well as conformity of the design concepts with the needs of SDT and Behavior Change Technique Taxonomy v1.

### 3.1. Development of behavior change strategies

#### 3.1.1. The importance of tailored feedback from a transparent sender

In the discussions during workshop 2, the participants agreed that it was important to receive feedback, reminders and notices because it indicated that their achievements were recognized. According to a few participants, just receiving a “thumps up” or a “smiley” was satisfactory. Most of the participants did however feel that the feedback needed to contain some personal relevance to be motivating. Both the discussions and the ranking of feedback messages showed that content that was experienced as meaningful was not the same for all of the participants. In the analyses, both content and wording of the messages as well as uncertainty of the identity of the sender was found to affect how the feedback was perceived.

The tone in the messages was considered to be important for how the content was interpreted. Only a limited number of the self-constructed feedback messages, each of which had a positive tone, were considered to be motivating according to all older adults. One of the messages “*Get up and get to it, lazy bones!”*, was highly appreciated by some, but others found it offensive. The participants expressed that when presented with feedback like this, the transparency of the sender became crucial. One of the participants said that it could be okay “*if it is directly towards a friend …//… you need to hear the tone”*. One woman said that a physiotherapist could say this *if* you are familiar with each other, otherwise it could be considered offensive. Through the discussion it became clear that when receiving a text message from an unknown person it was easy to misunderstand the intentions and interpret the content and tone it in multiple ways.

Another example of when the sender was unclear and the message was considered to be provocative is “*We've seen that you haven't worked out in a while. It's always a bit challenging in the beginning. Try some new exercises.”*. One senior said: “*I think that it is like that little pointer – I don't like/appreciate that. “WE have seen', “Big Brother is watching you!', Yes, you are supervised”*. The frustration and feeling of being observed arose when discussing feedback messages presented for non-performed activity. Messages related to activities which were performed according to plan were, on the other hand, often highly appreciated.

The content and wording of the message was considered to be important. Some messages were appreciated because the older adults believed and trusted their content, e.g., the message, “Research *suggests that you are more likely to do your exercises if you incorporate them into your weekly schedule”*. The intention of some of the messages was not clear for some participants, who became annoyed with the wording, e.g., in the message, “*You need to plan when and where you're to do your exercises”*. A few participants preferred the more direct formulation, but some became very annoyed because of the phrasing, “*You need to*”. They did not appreciate the thought of an unknown person telling them what they needed or did not need to do. The participants appreciated receiving feedback from both a friend and a health professional. One participant who argued that the relationship to the sender was important said: “*It depends on who you talk to. Is it a doctor you find unpleasant or someone you think is really nice? Or is it a friend you are afraid of or a friend you can talk freely with?”* A few participants argued that the same feedback was appreciated regardless of who the sender was.

#### 3.1.2. Conceptualizing a virtual health coach

To elaborate more on the role of the sender, the participants were asked to write narratives (home task 2) about how they interacted with and used an mHealth application. Several older adults wrote narratives about how they perceived feedback and guidance from a health professional or health coach through the application. To learn more about who the sender should be and the type of personality and characteristics the sender should have, the older adults were asked to brainstorm in workshop 3 what they found important in communication with health professionals. During the assignment, different personalities among health professionals were established, and three distinct personas were formulated: enthusiastic, information-oriented, and interested. Although the groups used different words to describe the personalities (e.g., knowledgeable and competent), the characteristics of the personalities created by the both groups were mainly the same. The participants expressed that even though they wanted a combination of the mentioned personalities it was clear that each participant had one personality they preferred. The older adults also co-created feedback messages tailored to the personality of each virtual health coach.

**Figure 2:**
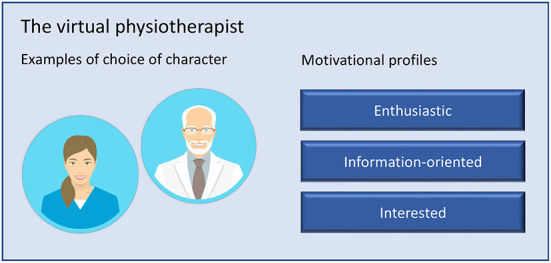
The virtual physiotherapist with options for tailoring of visualization and character.

As a result of the workshops, a virtual physiotherapist was developed based on the design concept of the virtual health coach. The virtual physiotherapist was integrated in the Safe Step application with the goal to increase relatedness as well as act encouraging during a digital fall prevention exercise intervention. A short persona description was created for each of the motivational profiles of the physiotherapist to guide the older adults in their choice when starting to use the application. To support competence, the virtual physiotherapist delivered feedback messages after the participants had registered their performed exercise in an exercise diary in the application. Reminders and encouragement were also sent as a push-notifications. To increase the experience of relatedness and increase transparency of the sender, the older adults could choose between five different avatars to represent the physiotherapist: two women and two men (one younger and one older for each sex) dressed in medical attire and a dog (Figure 2). The dog was included as a choice to evaluate whether the human avatar was an important feature to create relatedness.

**Figure 3:**
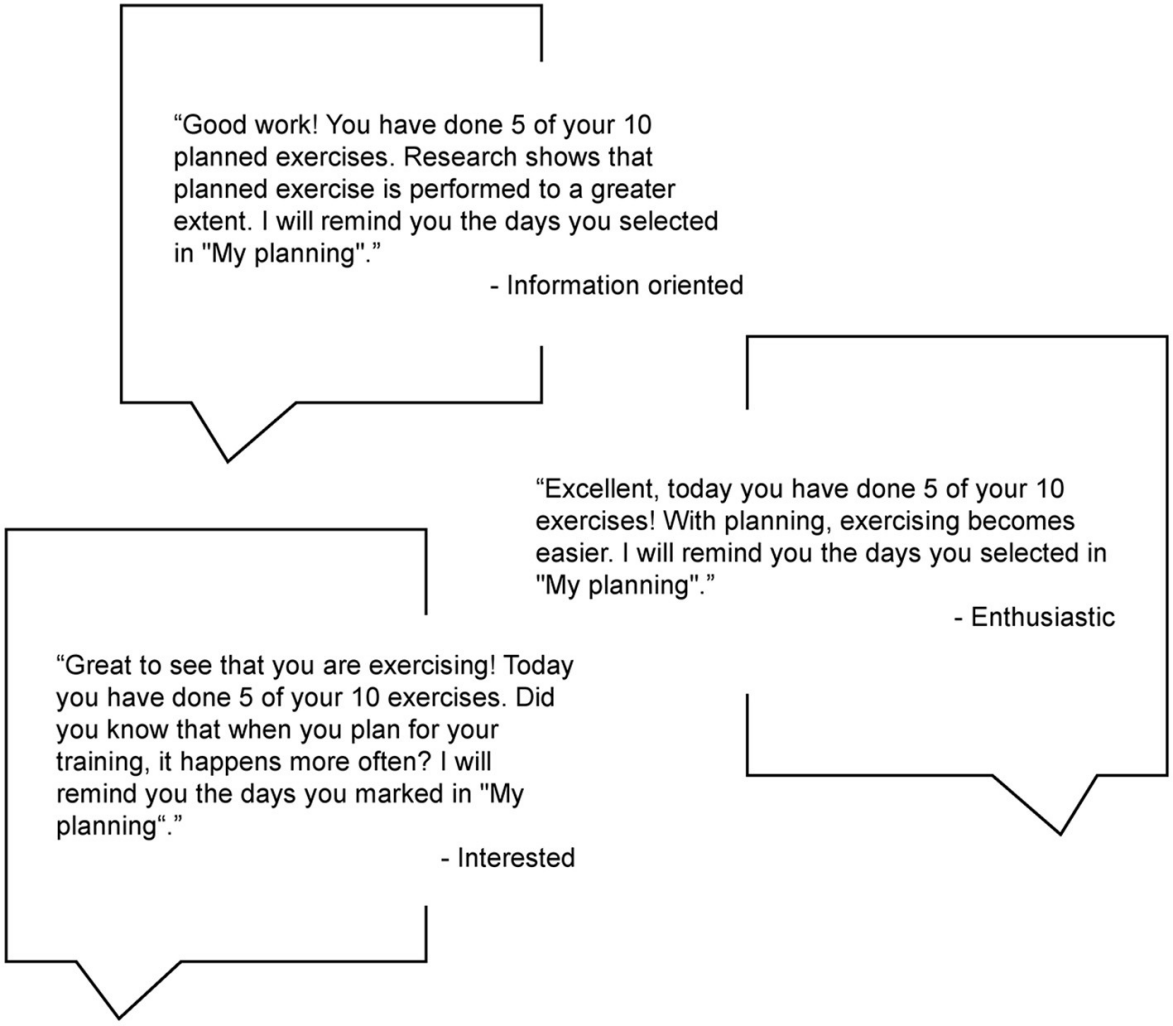
Examples of feedback messages delivered by the virtual physiotherapist, and the different motivational profiles, when half of the recommended exercise had been performed.

Each virtual physiotherapist used the same behavioral change techniques in the motivational messages, reminders, and notifications (Table 3). However, depending on the motivational profile the virtual physiotherapist applied a different set of motivational messages, reminders and encouragement. Tailoring the feedback aimed to help the older adults to relate to the virtual therapist and feel supported by it. The research group further developed the co-created messages by increasing the number of messages tailored to each virtual physiotherapist's personality to reduce the feeling of repetition. A total of 153 feedback messages were developed, 51 different messages per physiotherapist character. Messages were also developed to be based on the performed exercise and discrepancy between their own set goal of number of exercises for each session and the actual performed exercise; the messages continued with a sentence that acknowledged the particular performance in a positive way, regardless of number of exercises performed. The following sentence in the messages could be a suggestion to change exercises in the program, how to integrate the exercise in one's daily life, or why exercise is good for health (Figure 3).

**Table 3:** The table presents the behavior change strategies incorporated in the Safe Step-program v1 prototype according to the Behavior Change Techniques Taxonomy v1 (BCTTv1) (19), as well as their theoretical foundation from Self-determination Theory (SDT) (25).

**The Safe Step programme v1**	**BCTTv1, *code and name***	**SDT**
**Creating an exercise program**		
Participants are informed of a weekly training goal of at least 30 min, three times per week. Participants are asked to create their exercise program by selecting 10 exercises from pre-specified exercise categories.	1.1. Goal setting (behavior)	Autonomy
Instructions on how to perform the exercises are presented in videos by older adults, in a home setting, with simultaneous verbal instructions.	4.1. Instructions on how to perform the behavior 6.1. Demonstration of the behavior 8.3. Habit formation	Autonomy Competence
The participants are continuously reminded by the exercise videos to start at a lower level of repetitions, and increase repetitions until they can complete two sets of 10 repetitions.	8.7. Graded tasks	Competence
Participants perform exercises which can improve strength and balance.	12.6. Body changes	Competence
**Action planning**		
Participants are asked to plan their exercise in a weekly planning screen.	1.4. Action planning	Autonomy Competence
**Monitoring**		
Participants are asked to record their exercise in an exercise diary integrated in the Safe Step program.	2.3. Self-monitoring of behavior	Autonomy Competence
In the exercise diary, participants respond to the question: “How did you feel after you had completed your exercises?”, which is also presented in the statistical overview.	5.4. Monitoring of emotional consequences	Competence
The participants are continuously reminded in the exercise videos to be attentive of improvements in strength or balance while doing the exercises, and to progress exercises accordingly.	2.4. Self-monitoring of outcome(s) of behavior	Competence Autonomy
**Feedback**		
Participants using the application receive push-notices on planned days of exercise. Participants using the program through the website received the reminders when logging in to the program.	7.1. Prompts/cues	Competence
Positive and encouraging motivational messages are delivered by a virtual physiotherapist with three motivational profiles. The messages are received when activity is registered. They are adapted to whether participants met exercise goals, and draw attention to the discrepancy between the completed exercise and the goal. “You have done 6 of your 10 planned exercises”.	1.6. Discrepancy between current behavior and goal 3.1. Social support (unspecified) 10.4. Social reward 15.1. Verbal persuasion about capability	Competence Relatedness
Positive behavioral feedback is delivered by a virtual physiotherapist directly after exercise is registered. In the “My results” screen, participants can view a summary of their performed exercises and self-rated daily form based on their registered exercise.	2.2. Feedback on behavior	Competence
**Educational information**		
Information about accidental falls and fall prevention is presented in written text. Presentations within the Safe Step program of older adults' experiences of using the exercise program.	9.1. Credible source	Competence
A hyperlink to a Swedish TV segment was included in the Safe Step program. In the segment, an older adult who used the Safe Step program describes his positive experiences.	6.3 Information about others' approval	Relatedness
**Other**		
Suggestions regarding exercising in different contexts, i.e., while waiting for water to boil, or the bus to arrive. Instructional videos also suggested exercising outside.	8.2. Behavior substitution 8.6. Generalization of target behavior	Competence Autonomy

Rows in green represent strategies developed in the present study.

In order to support the individual's autonomy and competence, the virtual physiotherapist reminds the older adults to set goals for their exercise by making weekly exercise plans. This flexibility makes it possible for the user to adapt their physical activity plan to fit other activities in their life and feel in control of the activity.

#### 3.1.3. Setting goals, visualization of performance, and social support

In workshop 3, participants performed activities to brainstorm and discuss visualization of results and performance. Several of the older adults expressed that they wrote “to-do” lists on paper or in their calendars to summarize what they wanted or planned to do during the coming week. They also found pleasure in being able to see visually what they had accomplished during the previous week. Both groups expressed a need for being able to plan their weekly physical activity in an application and wanted to be able to verify what they had completed. One participant expressed: “*A checklist might be good, then you can check exercises off and perhaps also contemplate on why things did not get done”*.

The older adults agreed that they had no interest in comparing and relating their results to others. In fact, they found it stressful to see the achievements of others and reported that it could create a sense of failure. They, therefore, wanted to be able to see their performance in relation to their own set goals. One man expressed this as: “*It is not uninteresting to see others' performance [in the application], but the most important thing is what you do yourself. I don't have the same physical ability as many of my peers because I'm so heavy. I've always been heavy. For example, when I was doing military service, we ran a distance of maybe 300 meters...I was 100 meters behind. I couldn't keep up and everyone knew that.”* Therefore, elements of gamification such as receiving awards or competing with others were discussed in the workshop but not further elaborated on. Both groups wanted to present their performance in the form of statistics presented over weeks and months as they thought it would provide them with a useful overview. The older adults additionally expressed that they want to be able to see their performance alongside their perceived health status for the day, as they thought this would provide them with a more comprehensive and explanatory overview.

When integrating the design concepts into a prototype, the weekly plan and the registration of performed exercises were both designed as checklists according to the participants' expressed wishes in the workshops. The registration is completed by checking-off the performed exercises from the list. By checking-off, instead of answering “Yes - I have done the exercise” or “No - I haven't done the exercise” the focus is on performance instead of non-performance, which supports competence.

### 3.2. Classification of behavior change techniques and theoretical constructs

We used the Behavior Change Techniques Taxonomy v1 (19) to classify the developed behavior change strategies and show how they could be integrated in and complement an existing mHealth application. We identified that the developed strategies employed 9 out of 93 techniques, which mainly corresponded to techniques related to feedback, action planning and self-monitoring. The behavior change strategies were developed with SDT as a theoretical foundation. Therefore, Table 3 also shows which basic human psychological need the strategies aimed to satisfy. In some cases, we found that the strategies could support two constructs and therefore classified them as both.

## 4. Discussion

Through an iterative and theory-driven co-creation process, we have developed behavior change strategies for mHealth solutions to support physical activity for older adults. In the co-creation process, we found that the older adults could perceive the same feedback messages differently depending on who they believed the sender was. Based on the importance of transparency regarding the identification of the sender of feedback, a virtual coach who delivers the feedback messages was conceptualized. The feedback messages were constructed to always provide encouraging comments regardless of performance. A scoping review of physical activity messaging supports gain-framed messages when delivered to older adults, as well as emphasizing social and mental health benefits of physical activity (34). Based on the participants' comments, the messages were also positively framed to avoid feelings of being disciplined. This was done in order to strengthen both competence and autonomy. In confirmation of this finding, the use of a non-controlling language was previously found in a meta-analysis to be important for promoting autonomy satisfaction (27). Conversely, both provision of structure and provision of information was found to negatively predict autonomous motivation (27), which could be related to the manner in which the communication took place with a health-care provider. Collectively, these results emphasize the importance of being able to deliver positive feedback that is tailored to individual's preferences regarding communication and motivation.

In this study, we developed a virtual coaching approach in order to increase the transparency of the sender of feedback, but also to increase relatedness with the program. Virtual coaching can be used both with and without human involvement in sending instructions and feedback. Even though mHealth applications can be argued to be less intrusive than traditional interventions, it is important to build systems that are adaptable and/or adaptive to personal preferences and that aim for unobtrusiveness (35). The co-creation of the virtual physiotherapist resulted in three different motivational profiles as the participants had different preferences regarding the formulation of the messages. Similar findings of older adults' preferences for diverse and personalized messages have been presented in a feasibility study of a personalized physical activity intervention delivered *via* smartphone (36).

In further development, feedback could be tailored more toward individual preferences, goals, and type of motivation for physical activity (25, 37). The use of digital technology is expected to increase in health promotion over the coming years worldwide (38). In order to provide individually tailored behavior change strategies on a large scale, preventative interventions aimed at promoting physical activity for older adults need to utilize more advanced solutions for how to provide and tailor behavior change strategies. The advancement of techniques and theoretical frameworks in artificial intelligence (AI) allows for new approaches to automated tailoring of support to individual needs, preferences, motivation and situations (39–42), as well as in how the interaction is designed (40, 43, 44), for instance, using embodied avatars and natural language (45). The use of AI would allow automated tailoring of feedback and other supportive strategies to an initial motivational model, part of the system's user model, and continuous adaptation based on changes in, for example, physical status, goals, motivation, and situation. In relation to our study, the tailoring could be based on self-reported data from an integrated diary for physical activity or exercise in an application, but could also advantageously be combined with, e.g., sensor data from smartwatches that can provide data on physical activity, sedentary behavior, and exertion during activity. It is further important to embed techniques in the system for providing transparency and provide reasons for the advised exercises and messages that are tailored and delivered to the individuals. This can benefit both the health professional in verifying the knowledge embedded in the system and, the individual in developing trust in the system (46).

SDT was chosen as a theoretical framework in the development of the behavior change strategies due to its wide use and effectiveness in supporting physical activity behaviors (15, 47). The theory has also previously been found by the current research group to be suitable for exploring older adults' preferences and motivation for exercise (45). Among sedentary older adults, SDT-based counseling has also shown long-term effects of increased physical activity (48). The behavior change strategies developed in this study to promote self-managed physical activity among older adults are mainly focused on increased satisfaction of competence and autonomy. This supports both confidence and a feeling of mastery of the physical activity performance and support self-management of the physical activity according to the individuals' own goals and everyday living. Autonomy and competence have been found to be more strongly associated with physical activity and exercise maintenance than relatedness (47). However, a combination of need-supportive techniques that co-act with each other are suggested to be required to create a need-supportive environment (27). Therefore, the virtual coach was also created as an attempt to increase relatedness with the system. This expectation was confirmed in a qualitative evaluation of the behavior change support in the Safe step application (49). Experiences from a telecare intervention for older adults support the concept of relatedness with digital systems and suggest the utility of a broadened concept of relatedness beyond human-human interaction (50).

### 4.1. Strength and limitations

A strength of this study is inclusion of older adults in the development of the behavior change strategies. The importance of involving end-users in the design of mHealth technologies and technologies for older adults are continuously being recognized (23). A structured co-creation process can ensure the design of appealing technologies (23) and efficient interventions (51, 52). Another strength of this study is the description of the design process and the presentations of the behavior change support in relation to the Behavior Change Techniques Taxonomy v1 and the basic human psychological needs according to SDT. This will facilitate the evaluation of the behavior change strategies and comparison between studies promoting physical activity for older adults (18, 19).

A limitation of the developed feedback is that the messages could not be fully tailored to each individual. Tailored feedback has previously been associated with higher participant engagement (34). However, several other features were implemented to allow for tailoring to personal preferences and for automated tailoring by the system. First, when implemented in the Safe Step digital exercise program, the older adults could choose between three different motivational profiles of the virtual physiotherapist, which allowed tailoring of the feedback messages to personal preferences. Second, the feedback messages after registering exercise are based on performance, i.e., tailored based on the discrepancy of exercises performed and their own set goals. As mentioned, further research is warranted regarding tailoring and behavior change systems, in particular, to further understand the impact of different tailoring techniques (43).

It is noteworthy that the recruitment generated a group of older adults who were largely already active in group exercise, and can, therefore, be seen as already physically active. The participants were not asked to report hours per week of physical activity, and high and low levels of physical activity could therefore not be classified according to recommendation for physical activity for older adults (3). Nevertheless, the study aimed to design behavior change support for older adults' self-management of exercise in their own home without interaction of a health care professional or an instructor. The participants contribution to the design process is therefore still important as the own responsibility for initiating their exercise routine was not something that they were used to. The study also included older adults with limited use of digital technology. As the design process had an explorative focus, the varied experience of the participants was found valuable in developing the behavior change support. The authors, however, do acknowledge that it would have been of value to also include older adults who were more sedentary as this might have influenced the results of the design process.

## 5. Conclusion

This study was a participatory design process informed by theories of behavior change performed together with older adults. This approach was found highly useful for developing behavioral change strategies aimed at increasing physical activity adherence that could effectively be integrated in an existing mHealth application. More research is needed to further elucidate the importance of individual tailoring of the behavior change support as well as transparency of the systems.

## Data availability statement

The datasets presented in this article are not readily available because no transcripts are available in English. The data supporting the conclusions of this article will be made available by the authors, upon reasonable request. Requests to access the datasets should be directed to beatrice.pettersson@umu.se.

## Ethics statement

This study was reviewed and approved by Swedish Regional Ethical Review Board in Umeå. The patients/participants provided their written informed consent to participate in this study.

## Author contributions

RJ and BP performed the data collection, made the analyses, interpretation of data, and drafting of the manuscript. BP and MS were responsible for the integration of the design concepts in the Safe Step application. MS and HL made substantial contribution to the analysis and interpretation of the data, as well as critical revisions to the content of the manuscript. All authors read and approved the final manuscript. All authors conceptualized the research questions and the semi-structured interview guide.

## References

[1] Cunningham C Sullivan RO Caserotti P Tully MA. Consequences of physical inactivity in older adults: A systematic review of reviews and meta-analyses. Scand J Med Sci Sports. (2020) 30:816–27. 10.1111/sms.13616 32020713

[2] Sherrington C Fairhall NJ Wallbank GK Tiedemann A Michaleff ZA Howard K. Exercise for preventing falls in older people living in the community. Cochrane Database Syst Rev. (2019) 1:CD012424. 10.1002/14651858.CD012424.pub2 30703272 PMC6360922

[3] World Health Organization (WHO). WHO Guidelines on Physical Activity and Sedentary Behaviour. (2020). Geneva: World Health Organization. Available online at: https://www.who.int/publications/i/item/9789240015128 (accessed December 9, 2022).

[4] Harvey JA Chastin SF Skelton DA. How Sedentary are Older People? A Systematic Review of the Amount of Sedentary Behavior. J Aging Phys Act. (2015) 23:471–87. 10.1123/japa.2014-0164 25387160

[5] Jefferis BJ Sartini C Lee I-M Choi M Amuzu A Gutierrez C. Adherence to physical activity guidelines in older adults, using objectively measured physical activity in a population-based study. BMC Public Health. (2014) 14:382. 10.1186/1471-2458-14-382 24745369 PMC4021412

[6] Nyman SR Victor CR. Older people's participation in and engagement with falls prevention interventions in community settings: an augment to the Cochrane systematic review. Age Ageing. (2012) 41:16–23. 10.1093/ageing/afr103 21875865

[7] Mclaughlin M Delaney T Hall A Byaruhanga J Mackie P Grady A. (2021). Associations Between Digital Health Intervention Engagement, Physical Activity, and Sedentary Behavior: Systematic Review and Meta-analysis. J Med Internet Res. (2021) 23*:*e23180. 10.2196/23180 33605897 PMC8011420

[8] Mönninghoff A Kramer JN Hess AJ Ismailova K Teepe GW Car LT. Long-term effectiveness of mhealth physical activity interventions: systematic review and meta-analysis of randomized controlled trials. J Med Internet Res. (2021) 23:e26699. 10.2196/26699 33811021 PMC8122296

[9] Franco MR Tong A Howard K Sherrington C Ferreira PH Pinto RZ. Older people's perspectives on participation in physical activity: a systematic review and thematic synthesis of qualitative literature. Br J Sports Med. (2015) 49:1268–76. 10.1136/bjsports-2014-094015 25586911

[10] Mitzner TL Boron JB Fausset CB Adams AE Charness N Czaja SJ. Older adults talk technology: technology usage and attitudes. Comput Human Behav. (2010) 26:1710–21. 10.1016/j.chb.2010.06.020 20967133 PMC2956433

[11] The Swedish Internet Foundation. Svenskarna Och Internet. (2021). Available from: https://svenskarnaochinternet.se/rapporter/ (accessed December 9, 2022).

[12] The Swedish Internet Foundation. Svenskarna Och Internet 2020. In: Digitalt utanförskap 2020 Q1 (2020). Available from: https://svenskarnaochinternet.se/rapporter/ (accessed December 9, 2022).

[13] Romeo A Edney S Plotnikoff R Curtis R Ryan J Sanders I. Can Smartphone Apps Increase Physical Activity? Systematic review and meta-analysis. J Med Internet Res. (2019) 21:e12053. 10.2196/12053 30888321 PMC6444212

[14] Kirk MA Amiri M Pirbaglou M Ritvo P. Wearable technology and physical activity behavior change in adults with chronic cardiometabolic disease: a systematic review and meta-analysis. Am J Health Promot. (2019) 33:778–91. 10.1177/0890117118816278 30586996

[15] Gourlan M Bernard P Bortolon C Romain AJ Lareyre O Carayol M. Efficacy of theory-based interventions to promote physical activity. A meta-analysis of randomised controlled trials. Health Psychol Rev. (2016) 10:50–66. 10.1080/17437199.2014.981777 25402606

[16] Conroy DE Yang CH Maher JP. Behavior change techniques in top-ranked mobile apps for physical activity. Am J Preven Medi. (2014) 46, 649–652. 10.1016/j.amepre.2014.01.010 24842742

[17] Domin A Spruijt-Metz D Theisen D Ouzzahra Y Vögele C. Smartphone-Based Interventions for Physical Activity Promotion: Scoping Review of the Evidence Over the Last 10 Years. JMIR Mhealth Uhealth. (2021) 9:e24308. 10.2196/24308 34287209 PMC8339983

[18] Teixeira PJ Marques MM Silva MN Brunet J Duda JL Haerens L. (2020). Classification of techniques used in self-determination theory-based interventions in health contexts: An expert consensus study. Motiv Sci. 6:438. 10.1037/mot0000172

[19] Michie S Richardson M Johnston M Abraham C Francis J Hardeman W. The behavior change technique taxonomy (v1) of 93 hierarchically clustered techniques: building an international consensus for the reporting of behavior change interventions. Ann Behav Med. (2013) 46:81–95. 10.1007/s12160-013-9486-6 23512568

[20] Chen K Chan AHS. A review of technology acceptance by older adults. Gerontechnology. (2011) 10:1–12. 10.4017/gt.2011.10.01.006.0036412613

[21] Carter A Liddle J Hall W Chenery H. Mobile phones in research and treatment: Ethical guidelines and future directions. JMIR mHealth uHealth. (2015) 3:e4538. 10.2196/mhealth.4538 26474545 PMC4704925

[22] Lindsay S Jackson D Schofield G Olivier P. Engaging older people using participatory design. in Proceedings of the SIGCHI conference on human factors in computing systems. New York, NY: ACM (2012). p. 1199–208.

[23] Merkel S Kucharski A. Participatory Design in Gerontechnology: A Systematic Literature Review. Gerontologist. (2019) 59:E16–25. 10.1093/geront/gny034 29788319

[24] Bødker K Kensing F Simonsen J. Participatory IT Design: Designing for Business and Workplace Realities. Cambridge, MA: MIT press (2009).

[25] Ryan RM Deci EL. Self-determination theory and the facilitation of intrinsic motivation, social development and well-being. Am Psychol. (2000) 55:10. 10.1037/0003-066X.55.1.68 11392867

[26] Teas E Friedman E Amireault S. Purpose in life and personal growth: The unique and joint contribution of physical activity and basic psychological needs. Appl Psychol Health Well Being. (2022) 14:795–818. 10.1111/aphw.12347 35107871

[27] Gillison FB Rouse P Standage M Sebire SJ Ryan RM. A meta-analysis of techniques to promote motivation for health behaviour change from a self-determination theory perspective. Health Psychol Rev. (2019) 13:110–30. 10.1080/17437199.2018.1534071 30295176

[28] Kitzinger J. Qualitative research. Introducing focus groups. BMJ. (1995) 311:299–302. 10.1136/bmj.311.7000.299 7633241 PMC2550365

[29] Janols R Lindgren HA. Method for Co-Designing Theory-Based Behaviour Change Systems for Health Promotion E.F.f.M.I. (EFMI), Editor. Amsterdam, Netherlands: IOS Press. (2017). p. 368–72. 28423816

[30] Ghaye T Melander-Wikman A Kisare M Chambers P Bergmark U Kostenius C. (2008). Participatory and appreciative action and reflection (PAAR)—democratizing reflective practices. Reflect Pract. 9:361–97. 10.1080/14623940802475827

[31] Michie S Abraham C Whittington C McAteer J Gupta S. Effective techniques in healthy eating and physical activity interventions: a meta-regression. Health Psychol. (2009) 28:690–701. 10.1037/a0016136 19916637

[32] Sandlund M Lindgren H Pohl P Melander-Wikman A Bergvall-Kåreborn B Lundin-Olsson L. Towards a mobile exercise application to prevent falls: a participatory design process, in Technology, Rehabilitation and Empowerment of People with Special Needs, eds. Pareto L, Sharkey PM, Merrick J. Hauppauge, NY: Nova Science Publishers (2015).

[33] Lindgren H Lundin-Olsson L Pohl P Sandlund M. End Users Transforming Experiences into Formal Information and Process Models for Personalised Health Interventions, MIE (2014). 25160210

[34] Williamson C Baker G Mutrie N Niven A Kelly P. Get the message? A scoping review of physical activity messaging. Int J Behav Nutr Phys Act. (2020) 17:51. 10.1186/s12966-020-00954-3 32295613 PMC7160981

[35] Oinas-Kukkonen H Harjumaa M. Persuasive systems design: key issues, process model, and system features. Commun Assoc Inf Syst. (2009) 24:18. 10.17705/1CAIS.02428

[36] Mair JL Hayes LD Campbell AK Buchan DS Easton C Sculthorpe N. A personalized smartphone-delivered just-in-time adaptive intervention (JitaBug) to increase physical activity in older adults: mixed methods feasibility study. JMIR Form Res. (2022) 6:e34662. 10.2196/34662 35389348 PMC9030994

[37] Ryan RM Deci EL. Organismic Integration Theory: Internalization and the Differentiation of Extrinsic Motivation, in Self-determination Theory: Basic Psychological Needs in Motivation, Development, and wellness. (2017). Guilford Press: New York. p. 179–215.

[38] World Health Organization. Global Strategy on Digital Health 2020-2025. Geneva: World Health Organization (2021).

[39] Baskar J Janols R Guerrero E Nieves JC Lindgren HA. Multipurpose goal model for personalised digital coaching. In: PAAMS 2017. Berlin, Germany: Springer. (2017). 10.1007/978-3-319-70887-4_6

[40] Guerrero E Nieves JC Lindgren H. An activity-centric argumentation framework for assistive technology aimed at improving health. J. Argument Comput. (2016) 7:5–33. 10.3233/AAC-160004

[41] Janols R Guerrero E Lindgren HA. Pilot Study on Personalised Coaching to Increase Older Adults' Physical and Social Activities. In: Ambient Intelligence – Software and Applications – 8th International Symposium on Ambient Intelligence. (2017). p. 140–8.

[42] Lindgren H Guerrero E Janols R. Personalised persuasive coaching to increase older adults' physical and social activities: a motivational model. in PAAMS. (2017). p. 170–82.

[43] Akker H Jones V Hermens H. Tailoring real-time physical activity coaching systems: a literature survey and model. User Model User-adapt Interact. (2014) 24:351–92. 10.1007/s11257-014-9146-y

[44] Laranjo L Dunn AG Tong HL Kocaballi AB Chen J Bashir R. Conversational agents in healthcare: a systematic review. J Am Med Inform Assoc. (2018) 25:1248–58. 10.1093/jamia/ocy072 30010941 PMC6118869

[45] Sandlund M Pohl P Ahlgren C Skelton DA Melander-Wikman A Bergvall-Kåreborn B. Gender perspective on older people's exercise preferences and motivators in the context of falls prevention: a qualitative study. Biomed Res Int. (2018) 2018:6865156. 10.1155/2018/6865156 30112416 PMC6077582

[46] Nowak A Lukowicz P Horodecki P. Assessing artificial intelligence for humanity: will AI be the our biggest ever advance? or the biggest threat. IEEE Technol Soc Mag. (2018) 37:26–34. 10.1109/MTS.2018.2876105

[47] Teixeira PJ Carraça EV Markland D Silva MN Ryan RM. Exercise, physical activity, and self-determination theory: a systematic review. Int J Behav Nutr Phys Act. (2012) 9:78. 10.1186/1479-5868-9-78 22726453 PMC3441783

[48] Van Hoecke A-S Delecluse C Bogaerts A Boen F. The long-term effectiveness of need-supportive physical activity counseling compared with a standard referral in sedentary older adults. J Aging Phys Act. (2014) 22:186–98. 10.1123/japa.2012-0261 23628840

[49] Pettersson B Janols R Wiklund M Lundin-Olsson L Sandlund M. Older adults' experiences of behavior change support in a digital fall prevention exercise program: qualitative study framed by the self-determination theory. J Med Internet Res. (2021) 23:e26235. 10.2196/26235 34328438 PMC8367180

[50] Schwaninger I Frauenberger C Fitzpatrick G. Unpacking Forms of Relatedness around Older People and Telecare, in Companion Publication of the 2020 ACM Designing Interactive Systems Conference. Eindhoven, Netherlands (2020). p. 163–9.

[51] Leask CF Sandlund M Skelton DA Altenburg TM Cardon G Chinapaw MJM. Framework, principles and recommendations for utilising participatory methodologies in the co-creation and evaluation of public health interventions. Res Involv Engagem. (2019) 5:2. 10.1186/s40900-018-0136-9 30652027 PMC6327557

[52] Durand MA Carpenter L Dolan H Bravo P Mann M Bunn F. Do interventions designed to support shared decision-making reduce health inequalities? a systematic review and meta-analysis. PLoS ONE. (2014) 9:e94670. 10.1371/journal.pone.0094670 24736389 PMC3988077

